# Gene Editing Profiles in 94 CRISPR-Cas9 Expressing T_0_ Transgenic Tobacco Lines Reveal High Frequencies of Chimeric Editing of the Target Gene

**DOI:** 10.3390/plants11243494

**Published:** 2022-12-13

**Authors:** Guo-Qing Song, Grace Urban, John T. Ryner, Gan-Yuan Zhong

**Affiliations:** 1 Department of Horticulture, Plant Biotechnology Resource and Outreach Center, Michigan State University, East Lansing, MI 48824, USA; 2 Grape Genetics Research Unit, USDA-Agricultural Research Service, Geneva, NY 14456, USA

**Keywords:** CRISPR-Cas9, editing efficiency, gene editing, GUS

## Abstract

Chimeric editing is often reported in gene editing. To assess how the general chimeric editing is, we created a transgenic tobacco line carrying a marker, *beta-glucuronidase* gene (*gusA*), introduced a CRISPR-Cas9 editing vector into the transgenic tobacco line for knocking out *gusA*, and then investigated the *gusA* editing efficiencies in T0 and subsequent generations. The editing vector carried a Cas9 gene, which was driven by the cauliflower mosaic virus 35S promoter, and two guide RNAs, gRNA1 and gRNA2, which were driven by *Arabidopsis* U6 (AtU6) and U3 (AtU3) promoter, respectively. The two gRNAs were designed to knock out a 42-nucleotide fragment of the coding region of *gusA*. The editing vector was transformed into *gusA*-containing tobacco leaves using *Agrobacterium tumefaciens*-mediated transformation and hygromycin selection. Hygromycin-resistant, independent T_0_ transgenic lines were used to evaluate *gusA*-editing efficiencies through histochemical GUS assays, polymerase chain reactions (PCR), and next-generation sequencing of PCR amplicons. Profiles of targeted sequences of 94 T_0_ transgenic lines revealed that these lines were regenerated from non-edited cells where subsequent editing occurred and created chimeric-edited cells in these lines during or after regeneration. Two of them had the target fragment of 42 bp pairs of nucleotides removed. Detail analysis showed that on-target mutations at the AtU6-gRNA1 site and the AtU3-gRNA2 site were found in 4.3% and 77.7% of T_0_ transgenic lines, respectively. To overcome the issue of extremely low editing efficiencies in T_0_ lines, we conducted a second round of shoot induction from the chimeric line(s) to enhance the success of obtaining lines with all or most cells edited. The mutation profiles in T_0_ transgenic lines provide valuable information to understand gene editing in plant cells with constitutively expressed CRISPR-Cas9 and gRNAs.

## 1. Introduction

The clustered regularly interspaced short palindromic repeats (CRISPR)-associated protein 9 (Cas9) system uses the Cas9 endonuclease led by a guide RNA (gRNA) to target DNA sites through nucleotide base pairing and induce DNA double-strand breaks for short insertion/deletion mutations [1,2,3]. This system has become a very powerful gene editing tool and has been widely used for modifying genes in various plant species [4,5,6,7].

Among other requirements, a desirable gene editing system should allow the effective induction of on-target mutations with minimum occurrences of off-target changes. Mutation frequencies (e.g., percentages of the cells with mutations) are often used to describe gene editing efficiencies. Gene editing frequencies are also described as the percentage of T_0_ regenerants in which on-target editing is detected in all cells. As known from many editing studies, many T_0_ editing lines or regenerants were chimeric [7,8,9,10,11]. The development of a method for increasing the proportion of non-chimeric/chimeric T_0_ editing events is important for enhancing the effectiveness of a gene editing research project.

In a previous gene editing study in which we intended to knock out the reporter gene *beta-glucuronidase* (*gusA*) in *gusA* transgenic blueberry, we observed very low mutation frequencies in the T_0_ calli (<6% in the best callus cluster) [8]. While successfully edited plants were recovered through the second round of shoot regeneration from leaf explants of 10 selected T_0_ lines, the overall editing efficiency was low (~15%) [8]. In a recent effort to knock out a 10.5 kb transposon from the promoter region of a grape *MybA1* gene (*VvMybA1*), only one out of hundreds T_0_ regenerants screened was a non-chimeric edited line, suggesting an extremely low efficiency in the production of putative editing lines even in stable transgenic lines where Cas9 and gRNAs were constitutively expressed [9]. To investigate whether the low editing efficiency observed in the blueberry study was species-specific and whether the second round of regeneration would help enhance the recovery of non-chimeric edited plants, we transformed the same editing vector into tobacco and evaluated the *gusA* editing efficiencies in transgenic calli and lines in T_0_ and subsequent second round of regenerants. Because tobacco can easily be transformed, we were able to produce close to 100 independent T_0_ lines to evaluate by sequencing in this study. This study also benefited from using *gusA* as a target gene as it provides an excellent marker to discern edited, non-edited, or chimeric plants through histochemical GUS staining assay. Our results confirmed that most T_0_ transformants were chimeric for the target gene editing and the second round of regeneration was useful for increasing the chance of obtaining non-chimeric edited lines.

## 2. Results

**Figure 1:**
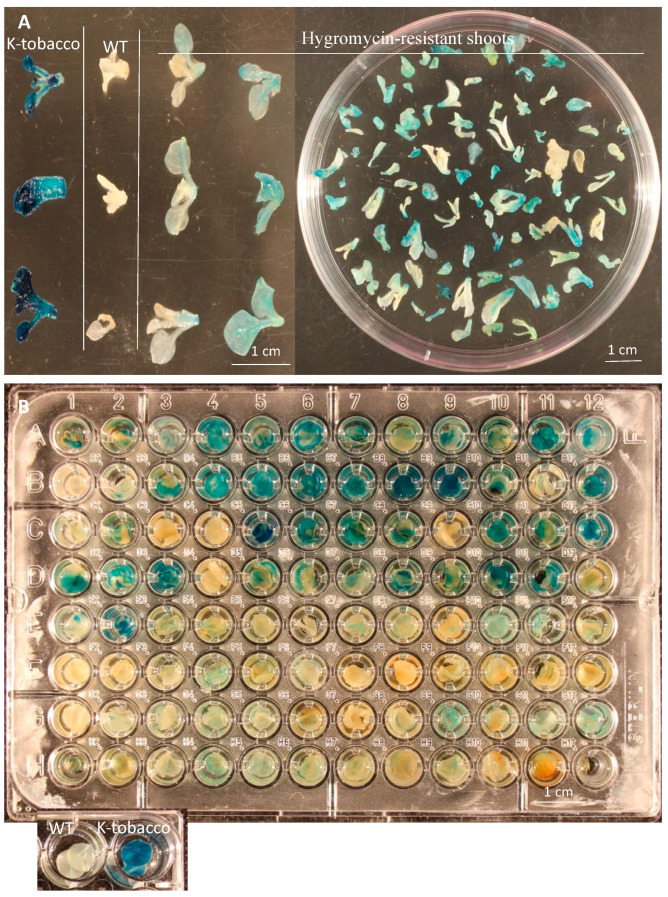
Histochemical GUS staining of tobacco tissues. (**A**) Hygromycin-resistant shoots each from different explants. (**B**) Leaf disks in individual wells were from 96 independent transgenic lines (hygromycin-resistant) containing the P35S-Cas9-GUS-gRNAs. WT: wild-type tobacco ‘Samsun’. K-tobacco: Kanamycin-resistant tobacco containing the pBISN1 vector with an active expressing *gusA*.

Hygromycin selection at 20 mg/L was very effective in inhibiting the regeneration of non-transformed cells. All transformed leaf disks produced hygromycin-resistant calli and shoots, and the shoots from different explants or different positions of the same explant were labeled as independent lines because there was barely any chance that they were from the same transformed cells. Histochemical GUS assay revealed that most hygromycin-resistant regenerants (hereafter: H-tobacco) showed a mixture of blue and white tissues, indicating that loss of the *gusA* function occurred in the transformed tissues due to the editing, as intense blue staining was observed in the shoots of kanamycin-resistant tobacco without transformation of the P35S-Cas9-GUS-gRNAs (hereafter: K-tobacco) (Figure 1A).

To evaluate the editing efficiencies of *gusA*, 2–3 individual hygromycin-resistant shoots from each leaf explant, selected 10 weeks after inoculation, were subjected to GUS staining. In two experiments, one had ~8.3% and the other had ~6.0% of the H-tobacco T_0_ lines producing shoots or leaf disks without visible blue staining (Figure 1), which likely resulted from knocking out the *gusA* gene. This agrees with 7/94 (7.4%) lines having over 98% editing efficiency for gRNA2 in experiment 2 (Table 1). About 50% of the regenerants in each experiment were likely chimeric with both edited and non-edited *gusA*, because they showed blue color much lighter than that in the K-tobacco tissues containing an active *gusA* in all cells. The tissues showing either no visible blue (presumably fully edited) or a partial blue (presumably chimeric) were not likely caused by poor penetration of the GUS staining solution because dark blue was shown in all control shoots, and the vacuum infiltration must have enabled well penetration of the GUS staining solution to cells.

The presence of *Cas9* and *hpt* in the H-tobacco transformants was verified using PCR before these transformants were subjected to PCR amplification of the *gusA* fragments covering the two targeted sites. Of the H-tobacco lines screened, we did not identify any lines showing only the single PCR band representing the target *gusA* ~42-bp fragment removed. This suggested that none of the transgenic lines were regenerated from a single edited cell with two target sites edited simultaneously. However, we did identify two transgenic lines which showed PCR bands corresponding to both edited and non-edited *gusA*, indicating that some cells had the target region removed in these two lines. Overall, the efficiency of simultaneously editing the two target sites was low.

To investigate the molecular features of *gusA* editing in the H-tobacco lines, sequences (~50 K reads per sample, Q > 30) of the PCR amplicons from newly developed leaf tissues of 94 randomly selected transgenic lines were produced and analyzed. Non-edited K-tobacco was used as a control (Table S1). For either gRNA1 or gRNA2, the K-tobacco control had insertion (Ins) and deletion (Del) (hereafter: Indel) frequencies less than 0.1%. We arbitrarily used Indel frequencies of above 3% as a criterion to define detectable edited transgenic lines for the analysis in this study.

**Figure 2:**
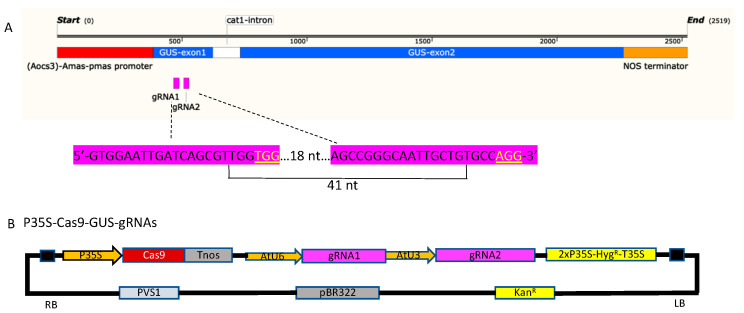
Schematic illustration of the editing target sites and vector. (**A**) *gusA* gene structure and gRNA target sites and sequences. GUS-gRNA80 and GUS-gRNA140 are marked in pink. The PAM sequences are underlined. (**B**) A schematic diagram of the editing vector. RB, right border; LB, left border; P35S, cauliflower mosaic virus 35S RNA gene promoter; AtU6, *A. thaliana* small RNA U6 promoter; AtU3, *A. thaliana* small RNA U3 promoter; T35S, cauliflower mosaic virus 35S terminator; Hyg^R^, hygromycin resistance gene; pVS1, replication origin from pseudomonas aeruginosa; pBR322, replication origin from pMB1; Kan^R^, kanamycin resistance gene.

For the gRNA1 site, 10.6% of the H-tobacco lines were edited and had both insertions and deletions—they were all chimeric. Of these chimeric lines, 16.1% of cells had deletions and 1.2% had insertions. Of the top Indels (> 1000 reads per mutation) in each edited transgenic line, most transgenic lines had 1-bp or 2-bp deletions and a 1-bp insertion with a thymine (“T”). 40-bp and 42-bp removal were the major large fragment deletions detected, which occurred in 1.1% and 2.1% of the transgenic lines, respectively (Table 2). However, there were a total of only three on-target mutations that occurred in four T_0_ H-tobacco lines (Table 2 and Table S1), including both 40-bp and 42-bp removal detected in three chimeric transgenic lines and one insertion line. The overall percentage of plants containing detectable on-target edited cells (editing frequency) was 4.3%. Notably, the 42-bp removal detected in two chimeric transgenic lines, which had a frequency of 36.5% and 2.6%, respectively, was an on-target deletion where gRNA1 and gRNA2 made cuts simultaneously (Figure 2A). Those off-target mutations for the gRNA1 were due mainly to the gRNA2.

For the gRNA2 site, there were a total of 10 H-tobacco lines which had over 90% of cells with editing at the gRNA2 target site (Table 2), while about 18% (17/94) of H-tobacco lines had mutation frequencies (<1%) similar to that of the K-tobacco control. Overall, 77.7% of the H-tobacco lines were edited, and seven (7.4%) transgenic lines showed editing frequencies greater than 98% (Table S1). Notably, all mutations detected for the gRNA2 site were on-target mutations (Table 2). Of these transgenic lines, 12.2% of cells had deletions, and 12.7% had insertions when sequences with a total of reads > 1000 for each line were included in the calculation (Table 2). Most of the transgenic lines had 1-bp (60.6%) or 2-bp (34.0%) deletions or a 1-bp (44.7%) insertion with thymine (“T”). A 40-bp deletion was detected in 2.1% (2/94) of the H-tobacco lines (Table 2). Interestingly, a single “T” insertion was the major form of insertion at both gRNA target sites. In fact, there was only one H-tobacco line showing an adenine (“A”) insertion, and there were no H-tobacco lines with detectable insertion of cytosine (“C”) or guanine (“G”). This does not seem to be a random event, because, in tomato, the most abundant insertion was “A” followed by “T”, “C”, and “G” [7]. Overall, the gRNA2 produced a higher number of edited transgenic lines than the gRNA1 (77.7% versus 4.3%).

We further checked profiles of the mutations in seven (7.4%) transgenic lines, each showing editing frequencies greater than 98%, and identified the major mutations with mutation frequencies greater than 10%. A total of 2–4 major mutations were found in each line, and none of these lines seemed to be produced from a single edited cell because over 20% of the Indels for each line were composed of multiple minor mutations (mutation frequencies < 10%) (Table 3). Apparently, chimeric editing of the target gene occurred in most, if not all, of the edited T_0_ lines, suggesting that the efficiency of obtaining non-chimeric edited T_0_ lines was very low.

To determine if GUS staining results were correlated with mutation frequencies detected by sequencing, we analyzed the staining and sequencing data from one K-tobacco and 94 H-tobacco lines (Figure 1B). A correlation analysis was conducted between the mutation frequency and the score of GUS staining for each line. The results showed little correlation (R^2^ = 0.0008), likely due to the chimeric nature of these transgenic lines (Figure S1). In other words, GUS staining in leaf disks was not a reliable criterion to determine whether an H-tobacco transformant was a non-chimeric edited or a chimeric line in the T_0_ generation because continuous editing is expected in the transgenic plants due to the Cas9 being driven by a constitutive promoter. Indeed, we observed that some samples had high mutation frequencies in young leaves while their older leaf disks still showed blue staining.

A second round of shoot regeneration experiments was performed by culturing leaf explants from different chimeric editing lines on the regeneration medium. When the induced young shoots were stained in GUS solution, it was obvious that more H-tobacco regenerants from the parent transgenic line with weak staining showed no blue staining than those from the parent line with intense staining (Figure 1A and Figure S1). The staining assay suggests that it is possible to increase the chance of obtaining lines from single Cas9-edited cells by conducting a second round of regeneration from chimeric-edited lines. This was further confirmed by the profiles of the mutations for the gRNA2 site in 30 transformants produced from six selected T_0_ transgenic lines from experiment #1 (Table 4). For the plants produced from three light blue lines containing presumably both edited and non-edited cells, ten out of 15 plants (66.7%) were non-chimeric edited lines. Of the 15 plants from three white T_0_ lines showing no GUS staining, they were all non-chimeric, and one plant with a 42-bp deletion was non-chimeric for both gRNA1 and gRNA2 sites (Table 4). The results demonstrate that a second round of regeneration from chimeric-edited lines can increase the potential for the generation of non-chimeric-edited plants.

## 3. Discussion

The phytoene desaturase (PDS) gene is often used in plant species as a candidate gene to determine gene editing efficiencies [13,14,15,16,17,18] because disruption of this gene causes albino leaves by impairing chlorophyll, carotenoid, and gibberellin biosynthesis [19,20]. However, spontaneous mutations can also cause albino tissues in plant regeneration, which may complicate PDS as a system to determine gene editing frequencies in some plants [21]. The *gusA* gene and the green fluorescent protein (*GFP*) gene are major screenable markers for plant genetic engineering. Both have been used in gene editing studies. For example, *gusA* was recently used to monitor the expression of the CRISPR-Cas9 [22] and was also used as an editing target for testing different gene editing platforms in a transgenic blueberry line [8]. In this study, we used *gusA* as an editing target for evaluating the efficiencies of obtaining edited lines in the T_0_ transgenic tobacco. As demonstrated, the GUS staining worked effectively in showing various types of editing outcomes in transgenic tobacco.

There are different ways to describe gene editing efficiencies. In editing studies using protoplasts, the mutation frequency is usually used to show the percentage of the edited and non-edited gene target(s). In editing studies using plant regenerants, the percentage of either plants/shoots or the molecules containing on-target editing is often used to indicate gene editing efficiency. In an editing study in transgenic tomato, the average mutation rate across 63 target genes in T_0_ was used as an estimate of editing efficiency [7]. The editing efficiencies for the two gRNA editing sites varied much in this study, with 10.6% for gRNA1 and 77.7% for gRNA2, although most of the edited plants were chimeric. The cause for the difference was unknown, but whether the AtU6 and AtU3 promoters had different strengths of promoter activities is an interesting question to examine. It was likely that the gRNA1 driven by the AtU6 promoter had a lower expression level than that of the gRNA2 driven directly by the AtU3 and indirectly by the upstream AtU6 promoter. As expected, the editing efficiency for targeting two editing sites simultaneously was detected in two transgenic lines. Even with a high editing efficiency of 77.7% for gRNA2, T_0_ transgenic plants regenerated initially from a single edited cell were not found in the 94 sequenced lines.

There are many factors, e.g., plant species, Cas9 sources, gRNAs, target cells, and editing approaches (i.e., error-prone nonhomologous end joining and homology-directed repair), that can affect gene editing frequencies [17,23,24,25]. This was supported by our sequencing data of 94 CRISPR-Cas9 expressing T_0_ transgenic tobacco lines, of which seven lines had over 98% of their cells edited at the gRNA2 site. Apparently, it is easier to regenerate plants from individual edited cells of these seven lines. To enhance the chance of obtaining non-chimeric edited plants, we demonstrated that a second round of regeneration would be very helpful. This information is important for those who are working on gene editing for clonally propagated plant species.

## 4. Materials and Methods

### 4.1. Plant Materials and Growth Conditions

The *gusA* vector, named pBISN1, was transformed into tobacco *Nicotiana tabacum* cv. Samsun for generating *gusA* transgenic tobacco lines. The vector contains a neomycin phosphotransferase II (*nptII*) gene as a selectable marker for kanamycin selection. The transformation was done following a published protocol (Duan et al. 2016). A T_0_ transgenic tobacco containing an active *gusA* was propagated on Murashige and Skoog (MS) medium containing 50 mg/L kanamycin [26] and used for *gusA* editing in this study (Murashige and Skoog 196). This T_0_ transgenic line showed one hybridization band in Southern Blot analysis and a ratio of transgenic to nontransgenic close to 3:1 in its first-generation seedlings. We considered this T_0_ transgenic line a line with a single copy of the transgenes. The cultures were maintained in our lab at 25 °C under a 16 h photoperiod of 30 µE m^−2^s^−1^ from cool white fluorescent tubes.

### 4.2. CRISPR-Cas9 Vector and Agrobacterium Tumefaciens-Mediated Transformation

CRISPR-Cas9 vector P35S-Cas9-GUS-gRNAs was constructed based on the previously described protocols [27]. The Cas9 was under the cauliflower mosaic virus 35S promoter (CaMV 35S). Two single gRNAs were chosen to target the *gusA* gene (Figure 2A), gRNA1-GUS80 (GTGGAATTGATCAGCGTTGG) and gRNA2-GUS121 (AGCCGGGCAATTGCTGTGCC). gRNA1 and gRNA2 were driven by AtU6 and AtU3 promoters, respectively. The two Cas9 target sites are 42 bp apart (Figure 2A). The binary vector P35S-Cas9-GUS-gRNAs has a hygromycin phosphotransferase (*hpt*) encoding resistance to hygromycin in plants and a kanamycin-resistant marker for bacteria (Figure 2B). It was transformed into *A. tumefaciens* strain EHA105 using the freeze–thaw method [28].

Leaf explants, ~0.8 cm × 0.8 cm, from in vitro cultured kanamycin-resistant, *gusA*-containing tobacco plants of a single-copy K-tobacco transgenic line were used for tobacco transformation by following a published protocol [29]. About 200 leaf disks were transformed and subject to selection in a medium containing 20 mg/L hygromycin in each of the two transformations conducted separately. The hygromycin-resistant shoots from separate explants were labeled as independent transgenic lines. They were grown on an MS medium containing 50 mg/L hygromycin.

To induce new shoot regeneration from selected T_0_ plants of the H-tobacco lines, leaf explants were cultured on a regeneration medium containing 50 mg/L hygromycin. Regenerated shoots were grown on an MS medium containing 50 mg/L hygromycin.

### 4.3. Histochemical GUS Staining Assay

Leaf disks/pieces or shoots were evaluated by histochemical GUS staining assay [30]. They were stained in 2 mM 5-bromo-4-chloro-3-indoyl-β-d-glucuronide (X-Gluc) (PhytoTech Labs, Overland Park, KS, USA) in 100 mM phosphate buffer for 24 h at 37 °C after a two-minute vacuum at 80,000 Pa, and chlorophyll was removed with 70% ethanol washes. The blue staining for each leaf disk was graded on a scale of 0 (no blue for wild-type control) to 4 (all blue for non-edited control). Three leaf disks/pieces for each plant of 100 randomly selected T_0_ lines were stained and scored; 95 of these lines were sampled for PCR amplificon sequencing.

### 4.4. DNA isolation and PCR Analysis

Approximately 100 mg leaf tissue for each sample was used for DNA isolation using the cetyltrimethylammonium bromide method [31]. Three primer pairs were used to identify transgenic calli or shoots by genomic PCR, including *hpt* F/R 5′-GCCTGAACTCACCGCGAC-3 and 5′-CGTCGGTTTCCACTARCGC-3′; *Cas9* F/R 5′-GGG TGA CCT TAA CCC TGA TAA C-3′ and 5′-CGA AAG TCC TCT GCT TCC TAA G-3′; and *gusA* F/R 5′-CGTACCTCGCATTACCCTTAC-3′ and 5′-AACGTATCCACGCCGTATTC-3′.

### 4.5. PCR Amplificon Sequencing and Identification of Edited Cells

Amplicon amplification was run as follows: the first round of PCR reaction was conducted to produce a GUS amplicon covering both Cas9 target sites with the GUS-specific primers-forward ATGTTACGTCCTGTAGAAA and reverse GCTCCATCACTTCCTGATTAT. The second PCR reaction was conducted to add adaptor sequences using the GUS-specific primers (upper case) with Illumina adaptor sequences (lower case): forward primer acactgacgacatggttctacaTCGTCCGTCCTGTAGAAA and reverse primer tacggtagcagagacttggtctGCTCCATCACTTCCTGATTAT. The PCR products were barcoded and sequenced using the Illumina platform in the RTSF Genomics Core of Michigan State University (https://rtsf.natsci.msu.edu/genomics/sample-requirements/illumina-sequencing-sample-requirements/) (accessed on 10 November 2022).

Sequencing reads were assessed using the FastQC program (https://www.bioinformatics.babraham.ac.uk/projects/fastqc/) (accessed on 10 November 2022), and high-quality reads with per base quality scores greater or equal to 30 were further analyzed to identify edited targets using the online Cas-Analyzer (http://www.rgenome.net/cas-analyzer/#!) (accessed on 10 December 2022) [12].

## 5. Conclusions

All T_0_ transgenic plants were regenerated before a gene editing event took place. Our sequence data lay a foundation that a second round of regeneration from T_0_ chimeric lines can increase the chance for the production of putative editing lines.

## Figures and Tables

**Table 1:** Estimated *gusA* editing frequencies based on the histochemical GUS staining of hygromycin-resistant T_0_ transgenic tobacco shoots transformed with the P35S-Cas9-GUS-gRNAs. Edited shoots or tissues refer to those that showed no visible blue staining.

Experiment	No. of Fully Edited Shoots (%)	No. of Chimeric Shoots (%)	No. of Non-Edited Shoots (%)	Total Number of Shoots
Experiment #1	50 (8.3%)	310 (51.8%)	239 (39.9%)	599
Experiment #2	12 (6.0%)	110 (55.0%)	78 (39.0%)	200

**Table 2:** Summary of PCR amplicon sequences from 73 *gusA* edited H-tobacco lines (Indel frequencies > 3%). The editing positions were identified using the online Cas-Analyzer (http://www.rgenome.net/cas-analyzer/#!) (accessed on 10 December 2022) [12]. For each line, sequences with a total of reads > 1000 were included in the calculation. % of T_0_ plants = number of H-tobacco T_0_ lines with the particular target sequence variant divided by 94 (total number of the sequenced T_0_ lines) × 100. Avg % of reads = Average percentage of reads out of the total reads from all edited lines that had the target sequence variant. Chance (%) of obtaining a non-chimeric edited variant = % of T_0_ plants × Avg % of cells × 100. This represents the chance of obtaining a non-chimeric edited line for the specific sequence variant in the second round of regeneration (given no continuous editing during the regeneration). WT: non-edited sequence. Del: Deletion. Ins: Insertion. Underlined letters show PAM sequences of the gRNAs.

Target Sequence Variant		% of T_0_ Plants	Avg % of Reads	Chance (%) of Obtaining Non-chimeric Editing
**gRNA1:** GTGGAATTGATCAGCGTTGGTGG				
CAGCGTTGGTGGGAAAGCGCGTTACAAGAAAGCCGGGCAATTGCTGTGCCAGG	WT			
CAGCGTTGGTGGGAAAGCGCGTTACAAGAAAGCCGGGCAATTGCT-TGCCAGG*	1-bp del	10.6	15.5	1.6
CAGCGTTGGTGGGAAAGCGCGTTACAAGAAAGCCGGGCAATTGC--TGCCAGG*	2-bp del	7.4	7.4	0.6
CAGCGTTGGTGGGAAAGCGCGTTACAAGAAAGCCGGGCAATT----TGCCAGG*	4-bp del	2.1	17.8	0.4
CAGCGTTGGTGGGAAAGCGCGTTACAAGAAAGCCGGGCAA------------------*	18-bp del	1.1	7.7	0.1
CAGCGT----------------------------------------TGCCAGG	40-bp del	1.1	33.0	0.4
CAGC-----------------------------------------TGCCAGG	42-bp del	2.1	22.7	0.5
	Del_all	10.6	16.1	1.7
CAGCGTTGGTGGGAAAGCGCGTTACAAGAAAGCCGGGCAATTTGCTGTGCCAGG*	1-bp ins	10.6	1.0	0.1
CAGCGTTGGTGGGAAAGCGCGTTACAAGAAAGCCGGGCAATATGCTGTGCCAGG*	1-bp ins	1.1	1.4	0.0
CAGCGTATGGTGGGAAAGCGCGTTACAAGAAAGCCGGGCAATTGCTGTGCCAGG	1-bp ins	1.1	5.2	0.1
	Ins_all	10.6	1.2	0.1
**gRNA2:** AGCCGGGCAATTGCTGTGCCAGG				
CAGCGTTGGTGGGAAAGCGCGTTACAAGAAAGCCGGGCAATTGCTGTGCCAGG	WT			
CAGCGTTGGTGGGAAAGCGCGTTACAAGAAAGCCGGGCAATTGCT-TGCCAGG	1-bp del	60.6	12.1	7.4
CAGCGTTGGTGGGAAAGCGCGTTACAAGAAAGCCGGGCAATTGC--TGCCAGG	2-bp del	34.0	10.3	3.5
CAGCGTTGGTGGGAAAGCGCGTTACAAGAAAGCCGGGCAATTG---TGCCAGG	3-bp del	1.1	16.3	0.2
CAGCGTTGGTGGGAAAGCGCGTTACAAGAAAGCCGGGCAATT----TGCCAGG	4-bp del	4.3	19.7	0.8
CAGCGTTGGTGGGAAAGCGCGTTACAAGAAAGCCGGGCAAT-----TGCCAGG	5-bp del	3.2	11.8	0.4
CAGCGTTGGTGGGAAAGCGCGTTACAAGAAAGCCGGGCAA------TGCCAGG	6-bp del	4.3	17.4	0.7
CAGCGTTGGTGGGAAAGCGCGTTACAAGAAAGCCGGGCAA----------------	16-bp del	2.1	16.6	0.4
CAGCGTTGGTGGGAAAGCGCGTTACAAGAAAGCCGGGCAA------------------	18-bp del	2.1	11.5	0.2
CAGCGTTGGTGGGAAAGCGCGTTACAAGAAAGCCGGGCAATT---------------------	21-bp del	1.1	8.0	0.1
CAGCGTTGGTGGGAAAGCGCGTTACAAGAAAGCCGGGCAATTGCT--------------------	30-bp del	1.1	2.8	0.0
CAGCGT----------------------------------------TGCCAGG	40-bp del	1.1	20.9	0.4
	Del_all	67.0	12.2	8.2
CAGCGTTGGTGGGAAAGCGCGTTACAAGAAAGCCGGGCAATTGCTGTTGCCAGG	1-bp ins	44.7	12.7	5.7
CAGCGTTGGTGGGAAAGCGCGTTACAAGAAAGCAGGGCAATTGCTGATGCCAGG	1-bp ins	1.1	0.7	0.0
	Ins_all	44.7	12.7	5.7

* Off-target editing for gRNA1.

**Table 3:** Profiles of major mutations in seven T_0_ transgenic lines each had an Indel frequency of over 98% for the gRNA2 site. WT: non-edited sequence.

Line	Mutation ID	Length	Indel# Count	Type	Inserted	No. of Deleted nt	Indel#	Indel Frequency
					Nucleotide (nt)			
S14							72,160	99.70%
	1	100	25,483	Del		40 *	25,483	35.3%
	2	141	10,142	Ins	T		10,142	14.1%
	3	139	10,021	Del		1	10,021	13.9%
	4	138	9687	Del		2	9687	13.4%
	5	140	171	WT			171	0.2%
				Other			16,656	23.1%
S7							39,237	99.6%
	1	138	9455	Del		2	9455	24.1%
	2	139	9379	Del		1	9379	23.9%
	3	141	8987	Ins	T		8987	22.9%
	4	140	96	WT			96	0.2%
				Other			11,320	28.9%
S66							62,155	99.5%
	1	99	24,126	Del		42*	24,126	38.8%
	2	139	13,189	Del		1	13,189	21.2%
	3	141	10,970	Ins	T		10,970	17.6%
	4	140	210	WT			210	0.3%
				Other			13,660	22.0%
S74							46,262	99.4%
	1	138	15,966	Del		2	15,966	34.5%
	2	134	8498	Del		6	8498	18.4%
	3	139	8320	Del		1	8320	18.0%
	4	140	221	WT			221	0.5%
				Other			13,257	28.7%
S49							36,465	99.1%
	1	141	12,902	Ins	T		12,902	35.4%
	2	139	7169	Del		1	7169	19.7%
	3	138	6464	Del		2	6464	17.7%
	4	140	221	WT			221	0.6%
				Other			9709	26.6%
S75							43,064	98.8%
	1	141	14,186	Ins	T		14,186	32.9%
	2	138	7608	Del		2	7608	17.7%
	3	134	6361	Del		6	6361	14.8%
	4	119	3081	Del		21	3081	7.2%
	5	140	327	WT			327	0.8%
				Other			11,501	26.7%
S93							33443	98.0%
	1	139	11,615	Del		1	11,615	34.7%
	2	141	10,135	Ins	T		10,135	30.3%
	3	138	1346	Del		2	1346	4.0%
	4	110	1060	Del		30	1060	3.2%
	5	140	501	WT			501	1.5%
				Other			8786	26.3%

* On-target deletion for both gRNA1 and gRNA2.

**Table 4:** Profiles of major mutations for the gRNA2 site in 30 transgenic plants produced through a second round of regeneration from six T_0_ transgenic lines, including three white lines without GUS staining and three blue lines with light blue color representing edited *gusA* camera. These T_0_ transgenic lines were from experiment #1, and profiles of the mutations in these lines were not available. WT: non-edited sequence. Del: Deletion. Ins: Insertion. Chimera: Mixture of various edited cells.

Line	Plant ID	Type	Inserted	No. of Deleted nt	Indel#	Indel Frequency
			Nucleotide (nt)			
White1						
	1	Del		1	162,884	96.6%
	2	Ins	T		234,945	96.1%
	3	Del		2	210,800	96.8%
	4	Del		1	159,958	96.8%
	5	Del		1	188,867	96.5%
White2						
	1	Ins	T		160,666	96.5%
	2	Del		1	195,805	96.4%
	3	Del		1	176,315	96.3%
	4	Del		42 *	195,712	99.9%
	5	Del		1	197,260	96.8%
White3						
	1	Del		1	111,402	96.6%
	2	Ins	T		110,705	96.6%
	3	Del		1	129,351	96.8%
	4	Ins	T		104,187	96.6%
	5	Del		1	108,926	97.1%
Blue1						
	1	Del		1	131,987	97.1%
	2	Del		1	126,944	96.6%
	3	Del		1	125,868	97.3%
	4	Del		1	110,997	97.0%
	5	Chimera			105,678	89.2%
Blue2						
	1	Ins	T		111,074	96.3%
	2	Chimera			125,305	96.5%
	3	Ins	T		125,601	96.6%
	4	Del		1	135,729	96.6%
	5	Chimera			117,808	87.7%
Blue3						
	1	Del		2	145,498	96.4%
	2	Del		1	143,450	95.9%
	3	Chimera			100,974	87.2%
	4	WT			77	0.0%
	5	Del		1	109,689	96.8%

* On-target deletion for both gRNA1 and gRNA2.

## Data Availability

The sequence data generated in this study are available from the corresponding author on reasonable request.

## Supplementary Materials

The following supporting information can be downloaded at: https://www.mdpi.com/article/10.3390/plants11243494/s1, Figure S1: Histochemical GUS staining of tobacco shoots regenerated from 8 selected leaf explants (A–H) which were hygromycin-resistant containing the P35S-Cas9-GUS-gRNAs. A total of 12 shoots (1–12) were randomly selected for staining; Table S1: Mutations identified in the PCR amplicon sequences from 94 *gusA* edited H-tobacco lines and one *gusA* containing non-edited K-tobacco. The editing positions were identified using the online Cas-Analyzer (http://www.rgenome.net/cas-analyzer/#!) (accessed on 10 December 2022) [12]. S91 is a K-tobacco without any editing.
