# Supplementary material for: Gene Editing Profiles in 94 CRISPR-Cas9 Expressing T0 Transgenic Tobacco Lines Reveal High Frequencies of Chimeric Editing of the Target Gene
Source: Plants (Basel). 2022 Dec 13;11(24):3494. doi: 10.3390/plants11243494 (PMC9782292; doi:10.3390/plants11243494)
Supplement: Supplementary file 1 [file plants-11-03494-s001.zip › plants-2057584-supplementary.pdf]

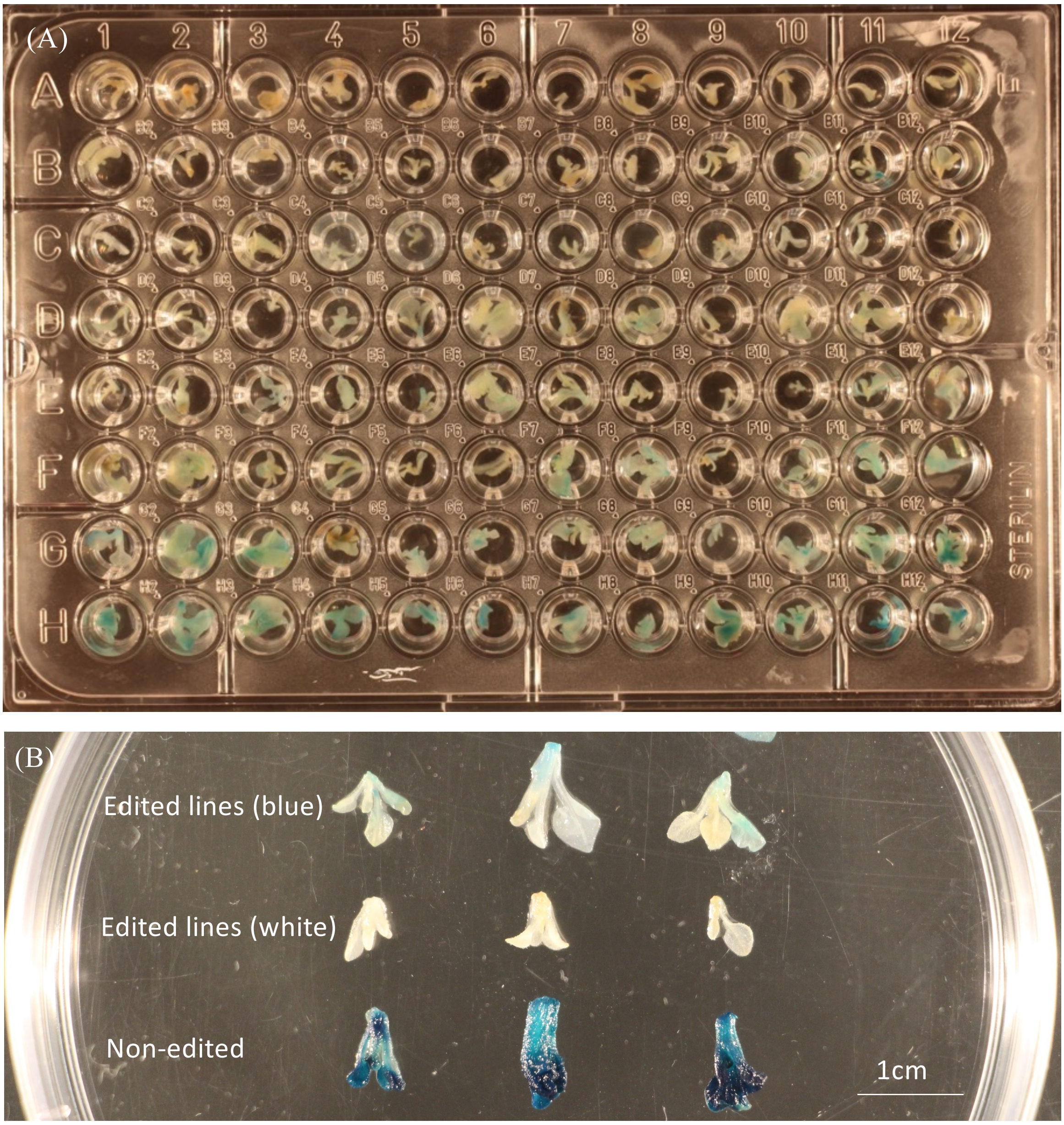

Figure S1 (A) Histochemical GUS staining of tobacco shoots regenerated from leaf explants of 8 (A-H) selected, hygromycin-resistant T_0 lines containing the P35S-Cas9-GUS-gRNAs. 12 shoots (1-12) randomly selected shoots were stained. (B) Examples of selected T_0 edited lines for second round regeneration and sequencing analysis.

Table S1 Mutations identified in the PCR amplicon sequences from 94 gusA edited H-tobacco lines and one gusA containing non-edited K-tobacco. The editing positions were identified using the online Cas-Analyzer (http://www.rgenome.net/cas-analyzer/#!) (Park, et al. 2017). S91 is a K-tobacco without any editing.

Sequence_id	gRNA	Total Sequences	With both indicator sequences	More than minimum frequency	Insertions	Deletions	Indel frequency
S89	Gus-gRNA1	78670	78234	74602	0	23	23 (0.0%)
S34	Gus-gRNA1	42041	41751	39588	0	13	13 (0.0%)
S42	Gus-gRNA1	44821	44547	42402	0	9	9 (0.0%)
S58	Gus-gRNA1	39780	39545	37624	0	15	15 (0.0%)
S35	Gus-gRNA1	24167	24021	22737	3	7	10 (0.0%)
S67	Gus-gRNA1	44173	43882	41661	0	14	14 (0.0%)
S91	**Gus-gRNA1**	**45792**	**45516**	**43230**	**0**	**12**	**12 (0.0%)**
S36	Gus-gRNA1	41027	40807	39194	0	7	7 (0.0%)
S44	Gus-gRNA1	39092	38788	36776	0	11	11 (0.0%)
S52	Gus-gRNA1	46239	45943	43551	0	16	16 (0.0%)
S37	Gus-gRNA1	38371	38088	35996	0	14	14 (0.0%)
S24	Gus-gRNA1	18806	18723	17753	0	0	0 (0.0%)
S40	Gus-gRNA1	44500	44261	42078	0	7	7 (0.0%)
S72	Gus-gRNA1	42615	42403	40466	0	5	5 (0.0%)
S80	Gus-gRNA1	44542	44298	42327	0	10	10 (0.0%)
S1	Gus-gRNA1	39727	39473	37470	9	26	35 (0.1%)
S26	Gus-gRNA1	70627	70259	67205	6	47	53 (0.1%)
S20	Gus-gRNA1	63773	63396	60339	5	76	81 (0.1%)
S92	Gus-gRNA1	39479	38288	36470	5	22	27 (0.1%)
S78	Gus-gRNA1	42169	41903	39661	2	28	30 (0.1%)
S94	Gus-gRNA1	41809	41556	39545	3	33	36 (0.1%)
S79	Gus-gRNA1	12104	11978	11137	4	3	7 (0.1%)
S48	Gus-gRNA1	38317	38079	36143	0	26	26 (0.1%)
S64	Gus-gRNA1	42975	42682	40524	13	17	30 (0.1%)
S43	Gus-gRNA1	43287	43054	40980	14	70	84 (0.2%)
S60	Gus-gRNA1	38822	38557	36541	8	74	82 (0.2%)
S21	Gus-gRNA1	28672	28053	26343	13	40	53 (0.2%)
S16	Gus-gRNA1	33785	33598	31691	7	54	61 (0.2%)
S12	Gus-gRNA1	40437	40171	38281	7	95	102 (0.3%)
S38	Gus-gRNA1	42099	41816	39524	19	114	133 (0.3%)
S5	Gus-gRNA1	37986	37241	34747	31	122	153 (0.4%)
S45	Gus-gRNA1	47979	47642	45090	21	138	159 (0.4%)
S61	Gus-gRNA1	35369	35054	32664	30	86	116 (0.4%)
S68	Gus-gRNA1	40022	39668	37606	25	165	190 (0.5%)
S13	Gus-gRNA1	51500	51221	48492	0	259	259 (0.5%)
S27	Gus-gRNA1	30820	30564	28622	33	141	174 (0.6%)
S59	Gus-gRNA1	42039	41691	39151	24	226	250 (0.6%)
S84	Gus-gRNA1	28662	28447	26755	19	152	171 (0.6%)
S22	Gus-gRNA1	59279	58974	55881	273	68	341 (0.6%)
S82	Gus-gRNA1	47387	47154	44499	82	219	301 (0.7%)
S4	Gus-gRNA1	36024	35743	33817	18	209	227 (0.7%)
S95	Gus-gRNA1	47639	47364	44977	32	278	310 (0.7%)
S9	Gus-gRNA1	51266	50917	48310	61	349	410 (0.8%)
S17	Gus-gRNA1	66661	65586	61698	136	364	500 (0.8%)
S65	Gus-gRNA1	34508	34116	31894	88	183	271 (0.8%)
S88	Gus-gRNA1	28358	28173	26506	12	197	209 (0.8%)
S33	Gus-gRNA1	35903	35618	33579	54	253	307 (0.9%)
S15	Gus-gRNA1	36349	36086	33927	15	288	303 (0.9%)

Sequence_id	gRNA	Total Sequences	With both indicator sequences	More than minimum frequency	Insertions	Deletions	Indel frequency
S47	Gus-gRNA1	24211	24083	22758	94	119	213 (0.9%)
S73	Gus-gRNA1	42651	42215	39199	115	267	382 (1.0%)
S46	Gus-gRNA1	20552	20403	19205	111	73	184 (1.0%)
S31	Gus-gRNA1	41618	41405	39289	0	429	429 (1.1%)
S10	Gus-gRNA1	45441	45159	42789	9	517	526 (1.2%)
S62	Gus-gRNA1	20855	20726	19388	11	226	237 (1.2%)
S63	Gus-gRNA1	36826	36512	34218	61	343	404 (1.2%)
S87	Gus-gRNA1	51336	51041	48509	4	587	591 (1.2%)
S25	Gus-gRNA1	47847	47413	44705	82	490	572 (1.3%)
S53	Gus-gRNA1	37999	37777	35357	29	416	445 (1.3%)
S81	Gus-gRNA1	39302	39044	36662	255	267	522 (1.4%)
S2	Gus-gRNA1	40777	40354	37990	257	307	564 (1.5%)
S29	Gus-gRNA1	40178	39914	37509	49	499	548 (1.5%)
S23	Gus-gRNA1	32187	32054	30232	12	436	448 (1.5%)
S30	Gus-gRNA1	43802	43535	41066	17	636	653 (1.6%)
S51	Gus-gRNA1	44761	44337	41883	302	411	713 (1.7%)
S6	Gus-gRNA1	32171	31949	30077	12	503	515 (1.7%)
S71	Gus-gRNA1	21947	21796	20407	229	115	344 (1.7%)
S83	Gus-gRNA1	38407	38112	35972	21	613	634 (1.8%)
S39	Gus-gRNA1	25728	25575	23964	48	382	430 (1.8%)
S93	Gus-gRNA1	35998	34398	32494	271	337	608 (1.9%)
S54	Gus-gRNA1	38666	38275	35995	36	663	699 (1.9%)
S32	Gus-gRNA1	35097	34770	32415	2	620	622 (1.9%)
S18	Gus-gRNA1	47781	47197	44357	76	812	888 (2.0%)
S3	Gus-gRNA1	50513	50037	47069	355	587	942 (2.0%)
S28	Gus-gRNA1	41223	40820	38261	83	695	778 (2.0%)
S55	Gus-gRNA1	37522	37141	34808	246	446	692 (2.0%)
S41	Gus-gRNA1	32462	32281	30035	186	455	641 (2.1%)
S57	Gus-gRNA1	33926	33384	31062	70	591	661 (2.1%)
S74	Gus-gRNA1	48684	48385	45796	0	997	997 (2.2%)
S77	Gus-gRNA1	36767	36483	34153	36	726	762 (2.2%)
S49	Gus-gRNA1	38489	38248	36074	404	399	803 (2.2%)
S86	Gus-gRNA1	40594	40238	37678	68	758	826 (2.2%)
S7	Gus-gRNA1	41366	41018	38695	267	570	837 (2.2%)
S50	Gus-gRNA1	41143	40902	38480	735	151	886 (2.3%)
S75	Gus-gRNA1	45635	41071	38804	389	542	931 (2.4%)
S69	Gus-gRNA1	46853	46308	43385	414	694	1108 (2.6%)
S70	Gus-gRNA1	39598	39256	36779	49	1123	1172 (3.2%)
S90	Gus-gRNA1	42361	41944	39174	117	1493	1610 (4.1%)
S76	Gus-gRNA1	39710	39138	36528	98	1536	1634 (4.5%)
S56	Gus-gRNA1	29478	29312	27364	1615	449	2064 (7.5%)
S19	Gus-gRNA1	44350	44012	40920	1047	2139	3186 (7.8%)
S11	Gus-gRNA1	45800	45187	42112	3523	940	4463 (10.6%)
S85	Gus-gRNA1	37912	37670	35075	6320	1063	7383 (21.0%)
S8	Gus-gRNA1	47639	47489	45546	580	13731	14311 (31.4%)
S14	Gus-gRNA1	74764	74405	71468	252	31822	32074 (44.9%)
S66	Gus-gRNA1	64424	64081	61688	267	29852	30119 (48.8%)
S34	Gus-gRNA2	42041	41847	40415	9	44	53 (0.1%)
S42	Gus-gRNA2	44821	44654	43198	8	46	54 (0.1%)
S91	**Gus-gRNA2**	**45792**	**45621**	**43938**	**15**	**50**	**65 (0.1%)**
S36	Gus-gRNA2	41027	40910	39796	5	29	34 (0.1%)

Sequence_id	gRNA	Total Sequences	With both indicator sequences	More than minimum frequency	Insertions	Deletions	Indel frequency
S52	Gus-gRNA2	46239	46060	44379	13	47	60 (0.1%)
S40	Gus-gRNA2	44500	44357	42859	10	36	46 (0.1%)
S72	Gus-gRNA2	42615	42502	41144	12	18	30 (0.1%)
S89	Gus-gRNA2	78670	78366	75697	25	94	119 (0.2%)
S58	Gus-gRNA2	39780	39657	38410	25	47	72 (0.2%)
S35	Gus-gRNA2	24167	24078	23196	19	34	53 (0.2%)
S67	Gus-gRNA2	44173	43991	42401	16	62	78 (0.2%)
S44	Gus-gRNA2	39092	38955	37497	21	45	66 (0.2%)
S24	Gus-gRNA2	18806	18750	18126	4	43	47 (0.3%)
S78	Gus-gRNA2	42169	41973	40475	44	99	143 (0.4%)
S26	Gus-gRNA2	70627	70440	68453	110	366	476 (0.7%)
S80	Gus-gRNA2	44542	44412	43155	95	216	311 (0.7%)
S94	Gus-gRNA2	41809	41638	40160	102	214	316 (0.8%)
S92	Gus-gRNA2	39479	38346	37010	122	349	471 (1.3%)
S48	Gus-gRNA2	38317	38212	36825	243	473	716 (1.9%)
S64	Gus-gRNA2	42975	42845	41325	257	568	825 (2.0%)
S1	Gus-gRNA2	39727	39635	38310	254	632	886 (2.3%)
S37	Gus-gRNA2	38371	38143	36623	240	613	853 (2.3%)
S20	Gus-gRNA2	63773	63568	61495	525	1699	2224 (3.6%)
S13	Gus-gRNA2	51500	51302	49301	376	1992	2368 (4.8%)
S59	Gus-gRNA2	42039	41829	40027	581	1420	2001 (5.0%)
S12	Gus-gRNA2	40437	40298	38988	590	2013	2603 (6.7%)
S27	Gus-gRNA2	30820	30675	29238	789	1332	2121 (7.3%)
S43	Gus-gRNA2	43287	43112	41721	100	3288	3388 (8.1%)
S68	Gus-gRNA2	40022	39851	38372	689	2574	3263 (8.5%)
S79	Gus-gRNA2	12104	12001	11342	631	353	984 (8.7%)
S88	Gus-gRNA2	28358	28235	26992	373	2106	2479 (9.2%)
S16	Gus-gRNA2	33785	33683	32308	931	2358	3289 (10.2%)
S60	Gus-gRNA2	38822	38684	37299	830	3072	3902 (10.5%)
S45	Gus-gRNA2	47979	47832	45793	1197	3996	5193 (11.3%)
S21	Gus-gRNA2	28672	28159	26787	710	2452	3162 (11.8%)
S84	Gus-gRNA2	28662	28528	27226	537	2763	3300 (12.1%)
S4	Gus-gRNA2	36024	35800	34422	1296	2959	4255 (12.4%)
S38	Gus-gRNA2	42099	41978	40351	1583	3457	5040 (12.5%)
S9	Gus-gRNA2	51266	51125	49299	1913	4401	6314 (12.8%)
S5	Gus-gRNA2	37986	37390	35495	1402	5026	6428 (18.1%)
S61	Gus-gRNA2	35369	35166	33349	1883	4833	6716 (20.1%)
S54	Gus-gRNA2	38666	38517	36832	1827	5578	7405 (20.1%)
S82	Gus-gRNA2	47387	47251	45155	5141	4954	10095 (22.4%)
S70	Gus-gRNA2	39598	39467	37558	2052	7470	9522 (25.4%)
S46	Gus-gRNA2	20552	20456	19523	4708	358	5066 (25.9%)
S22	Gus-gRNA2	59279	59084	56938	14224	965	15189 (26.7%)
S65	Gus-gRNA2	34508	34386	32734	3092	5762	8854 (27.0%)
S15	Gus-gRNA2	36349	36167	34592	291	9901	10192 (29.5%)
S47	Gus-gRNA2	24211	24125	23048	221	6581	6802 (29.5%)
S33	Gus-gRNA2	35903	35769	34281	2539	7752	10291 (30.0%)
S73	Gus-gRNA2	42651	42434	40168	4899	7223	12122 (30.2%)
S17	Gus-gRNA2	66661	66241	63368	5891	13895	19786 (31.2%)
S28	Gus-gRNA2	41223	41100	39204	2982	9268	12250 (31.2%)
S95	Gus-gRNA2	47639	47489	45546	580	13731	14311 (31.4%)
S25	Gus-gRNA2	47847	47659	45761	3666	10829	14495 (31.7%)
S18	Gus-gRNA2	47781	47553	45396	3944	12014	15958 (35.2%)

Sequence_id	gRNA	Total Sequences	With both indicator sequences	More than minimum frequency	Insertions	Deletions	Indel frequency
S76	Gus-gRNA2	39710	39472	37455	3213	10590	13803 (36.9%)
S83	Gus-gRNA2	8407	38254	36589	1037	13445	14482 (39.6%)
S57	Gus-gRNA2	33926	33783	32032	3870	9177	13047 (40.7%)
S63	Gus-gRNA2	36826	36595	34821	2280	13112	15392 (44.2%)
S55	Gus-gRNA2	37522	37259	35511	11194	4784	15978 (45.0%)
S11	Gus-gRNA2	45800	45522	43099	4339	16597	20936 (48.6%)
S31	Gus-gRNA2	41618	41470	39873	100	20174	20274 (50.8%)
S85	Gus-gRNA2	37912	37771	35399	4203	14629	18832 (53.2%)
S3	Gus-gRNA2	50513	50290	48016	16212	9633	25845 (53.8%)
S8	Gus-gRNA2	41859	41639	39766	2649	19184	21833 (54.9%)
S86	Gus-gRNA2	40594	40370	38316	3039	18390	21429 (55.9%)
S10	Gus-gRNA2	45441	45295	43595	777	24691	25468 (58.4%)
S62	Gus-gRNA2	20855	20793	19784	231	12018	12249 (61.9%)
S53	Gus-gRNA2	37999	37867	35923	1788	21037	22825 (63.5%)
S81	Gus-gRNA2	39302	39164	37341	12866	10963	23829 (63.8%)
S2	Gus-gRNA2	40777	40584	38919	13535	12082	25617 (65.8%)
S32	Gus-gRNA2	35097	34863	33088	281	21563	21844 (66.0%)
S90	Gus-gRNA2	42361	42175	29	3895	22758	26653 (66.6%)
S87	Gus-gRNA2	51336	51127	49133	357	32684	33041 (67.2%)
S41	Gus-gRNA2	32462	32377	30490	11543	9019	20562 (67.4%)
S29	Gus-gRNA2	40178	40008	38115	2602	23503	26105 (68.5%)
S30	Gus-gRNA2	43802	43589	41856	530	28351	28881 (69.0%)
S51	Gus-gRNA2	44761	44540	42749	16325	15817	32142 (75.2%)
S23	Gus-gRNA2	32187	32099	30660	110	23417	23527 (76.7%)
S6	Gus-gRNA2	32171	32065	30672	386	24457	24843 (81.0%)
S39	Gus-gRNA2	25728	25639	24393	3382	16905	20287 (83.2%)
S19	Gus-gRNA2	44350	44151	41708	14768	20360	35128 (84.2%)
S56	Gus-gRNA2	29478	29388	27850	7249	16553	23802 (85.5%)
S69	Gus-gRNA2	46853	46487	44119	18431	20635	39066 (88.5%)
S77	Gus-gRNA2	36767	36592	34815	2089	29464	31553 (90.6%)
S50	Gus-gRNA2	41143	40954	39233	34583	1160	35743 (91.1%)
S71	Gus-gRNA2	21947	21835	20725	12687	6670	19357 (93.4%)
S93	Gus-gRNA2	35998	35873	34134	14176	19267	33443 (98.0%)
S75	Gus-gRNA2	45635	45405	43569	19661	23403	43064 (98.8%)
S49	Gus-gRNA2	38489	38341	36795	17864	18601	36465 (99.1%)
S74	Gus-gRNA2	48684	48457	46555	36	46226	46262 (99.4%)
S66	Gus-gRNA2	64424	64172	62466	14642	47513	62155 (99.5%)
S7	Gus-gRNA2	41366	41159	39388	12770	26467	39237 (99.6%)
S14	Gus-gRNA2	74764	74574	72405	13837	58323	72160 (99.7%)
